# Transcriptome analysis of activated sludge microbiomes reveals an unexpected role of minority nitrifiers in carbon metabolism

**DOI:** 10.1038/s42003-019-0418-2

**Published:** 2019-05-13

**Authors:** Yuya Sato, Tomoyuki Hori, Hideaki Koike, Ronald R. Navarro, Atsushi Ogata, Hiroshi Habe

**Affiliations:** 1 Environmental Management Research Institute, National Institute of Advanced Industrial Science and Technology, 16-1 Onogawa, Tsukuba, Ibaraki 305-8569 Japan; 2 Bioproduction Research Institute, National Institute of Advanced Industrial Science and Technology, 1-1-1 Higashi, Tsukuba, Ibaraki 305-8565 Japan

**Keywords:** Metagenomics, Microbial ecology, Water microbiology

## Abstract

Although metagenomics researches have illuminated microbial diversity in numerous biospheres, understanding individual microbial functions is yet difficult due to the complexity of ecosystems. To address this issue, we applied a metagenome-independent, de novo assembly–based metatranscriptomics to a complex microbiome, activated sludge, which has been used for wastewater treatment for over a century. Even though two bioreactors were operated under the same conditions, their performances differed from each other with unknown causes. Metatranscriptome profiles in high- and low-performance reactors demonstrated that denitrifiers contributed to the anaerobic degradation of heavy oil; however, no marked difference in the gene expression was found. Instead, gene expression-based nitrification activities that fueled the denitrifiers by providing the respiratory substrate were notably high in the high-performance reactor only. Nitrifiers—small minorities with relative abundances of <0.25%—governed the heavy-oil degradation performances of the reactors, unveiling an unexpected linkage of carbon- and nitrogen-metabolisms of the complex microbiome.

## Abstract (web summary)

Yuya Sato et al. present de novo RNA sequencing of microbial communities from two activated sludge bioreactors that differ in their heavy-oil degradation performance. They find that the higher performing bioreactor showed higher expression of nitrification genes, even though nitrifiers represent a small minority of the community.

## Introduction

Understanding the individual microbial roles in ecosystems is a great challenge in microbial ecology, because community function is expressed as the sum of the metabolic activities and interactions of various microbes^1–3^. A powerful tool to address this issue is metatranscriptome analysis;^4^ however, its capability and reliability are reduced by an insufficiency of reference metagenome sequences^5^. Accordingly, the roles of rare microorganisms tend to be masked, which is problematic because functional importance does not always correspond to population abundance^6–8^. Recently, de novo assembly–based transcriptome analysis (de novo RNA-seq) was developed to overcome the dependency on reference genome data size^9^. Here, we applied de novo RNA-seq to decipher individual microbial functions in the complex ecosystem of activated sludge^10^.

**Fig. 1:**
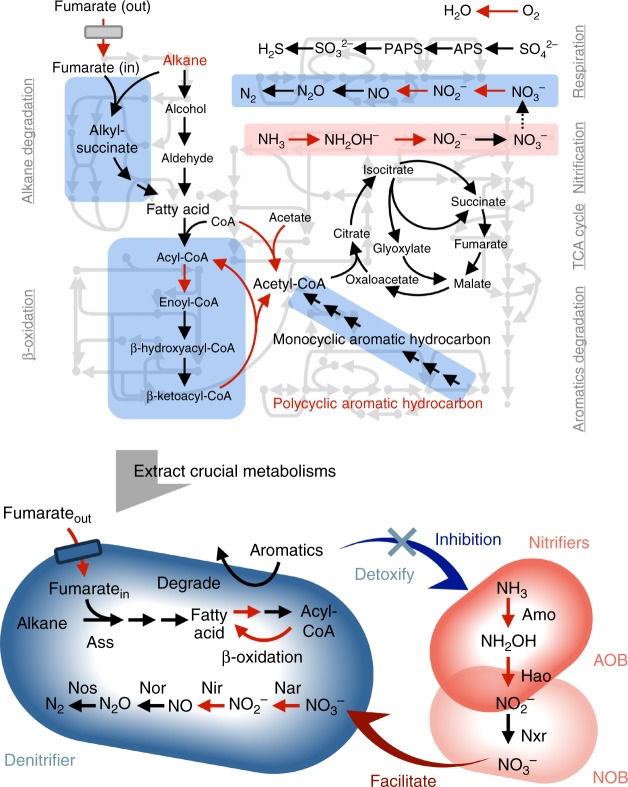
Heavy-oil degradation model in the sludge microbiome. Because the complete metabolic map of the ecosystem was unduly complex, several vital metabolic pathways were extracted by evaluating the microbial transcript diversity of the expressed genes (i.e., the number of microbial species expressing a gene) as well as the expression level (upper figure; Supplementary Table 2). On the basis of the extracted pathways, we proposed heavy-oil degradation mechanisms in the sludge microbiome, in which denitrifiers and nitrifiers indirectly cooperate. Red arrows indicate the metabolic pathways highly enriched. Blue and red boxes emphasize pathways possibly performed by denitrifier and nitrifiers, respectively. Gray lines in the background denote the representatives of metabolic pathways that were common but not directly related to the targeted key metabolisms. The lower figure shows the predicted interaction between denitrifier and nitrifiers. AOB and NOB denote ammonia-oxidizing bacteria and nitrite oxidizing bacteria, respectively

Even though activated sludge bioreactors have been used for wastewater treatment for over a century, the complexity of the sludge microbiome has hindered our precise understanding of the microbial processes (Fig. 1)^3,10^. It is difficult to stably manage the microbiomes in bioreactors, in part because of the unpredictable behaviors commonly observed in such complex microbial ecosystems^11^. Therefore, bioreactor performance occasionally deteriorates because of unknown causes; such problems are currently solved by impromptu means. Industrial and domestic wastewaters are often contaminated by heavy oil, which inhibits microbial activities and decreases reactor performance^12^. Although the degradation of heavy oil, which contains toxic aromatic hydrocarbons, by cultured microorganisms has been well studied^13,14^, the mechanisms underlying the degradation by complex microbiomes remain largely unknown. Here, we focused on membrane bioreactor (MBR), which is a representative activated sludge bioreactor^15^. The two replicate bioreactors were run under the same operational conditions for 37 days, during which heavy oil was spiked-in with increasing concentrations from day 20; yet, their performances became different from each other. We investigated these two reactors, one (reactor 1) with high heavy-oil degradation activity and one (reactor 2) with low activity, by metatranscriptome analysis based on de novo transcript assembly, referred to as de novo RNA-seq^9^. The results suggested that the small but important minorities of nitrifiers governed the heavy-oil degradation activities of the activated sludge bioreactors.

## Results

### Activated sludge reactor performance

**Fig. 2:**
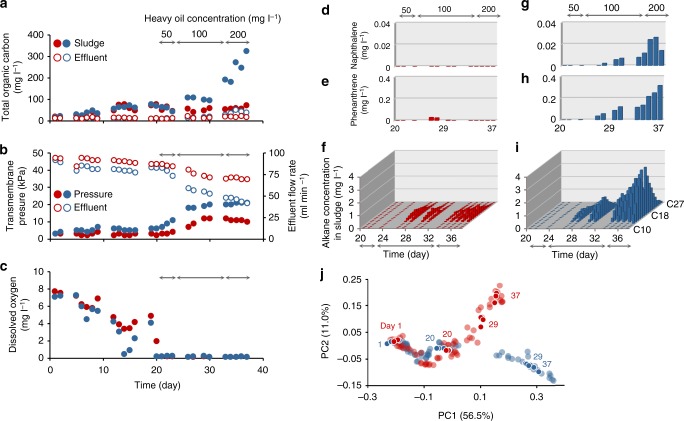
Dynamics of reactor performance parameters and microbial community structures in the two reactors. **a** Total organic carbon; **b** transmembrane pressure and effluent flow rate; **c** concentration of dissolved oxygen in sludge; and **d**–**i** concentrations of the following hydrocarbons in sludge: **d**, **g** naphthalene; **e**, **h** phenanthrene; **f**, **i**, alkane. Displayed hydrocarbon concentrations are mean values of duplicate measurements. **j** Scatter plot of principle-coordinate-analysis of 16S rRNA gene sequences conducted on the basis of the weighted UniFrac distance, generated by QIIME^32^. Distances between points denote similarity of microbial community structure, with smaller distances indicating greater similarity. Foreground points denote days 1, 20, 29, and 37, which correspond to start of operation and three time-points of RNA-seq analyses, respectively. For all panels, red and blue symbols or bars indicate reactors 1 and 2, respectively

As the inlet heavy-oil concentration increased (0, 50, 100, 200 mg l^−1^ at days 0–20, 21–23, 24–33, and 34–37, respectively), total organic carbon levels and concentrations of alkanes (C14–27) and polycyclic aromatic hydrocarbons (PAHs; naphthalene and phenanthrene) increased in reactor 2, but not in reactor 1 (Fig. 2a, d–i). Estimated alkane- and PAH-removal ratios in reactor 1 were above 90% throughout the operation, whereas those in reactor 2—especially for heavier alkanes—had decreased to around 60% by day 37 (Supplementary Fig. 1a–g), indicating lower heavy-oil degradation activity in the latter. During this period, transmembrane pressure increased and the effluent flow rate decreased in both reactors, but the magnitude of change was greater in reactor 2 (Fig. 2b). Sixteen-S ribosomal RNA (rRNA) gene amplicon sequencing demonstrated similar microbial community structures in the two reactors during the first 20 days; however, they became distinct after heavy-oil addition (Fig. 2j, Supplementary Figs. 1h and 2). Although differences in the dominant microorganisms in the two reactors were observed, the microbial ecophysiological information was not sufficient to elucidate the heavy-oil–degradation mechanism; therefore, we conducted additional de novo RNA-seq analyses.

### De novo assembly of transcripts

Six double-stranded complementary DNA (cDNA) libraries of gene transcripts were prepared by using RNAs extracted from the two reactors on days 20, 29, and 37. The scheme of the sequence analysis is summarized in Supplementary Fig. 3, and the results are listed in Supplementary Table 1. De novo assembly of the sequence data using Trinity generated 23,709 and 20,677 transcript assemblies for reactors 1 and 2, respectively^9^. We first focused on rRNA expression levels (Supplementary Fig. 4). Relative expression levels of the class Betaproteobacteria were abundant in the two reactors and increased after heavy-oil addition (Supplementary Fig. 4a, b). Genus-level analysis of Betaproteobacteria exhibited the similar dominant members as those revealed by DNA-based analysis (Supplementary Figs. 2c, i and 4c, d). Because rRNA, transfer RNA (tRNA) and transfer-messenger RNA (tmRNA) accounted for approximately 91%, 0.16% and 1.3%, respectively, of total gene expression^16^, TPM values (Transcripts Per kilobase Million representing relative abundances of transcripts) for the coding sequence (CDS) analysis were normalized without rRNA, tRNA, or tmRNA assemblies.

### Heavy-oil degradation mechanism in sludge microbiome

**Fig. 3:**
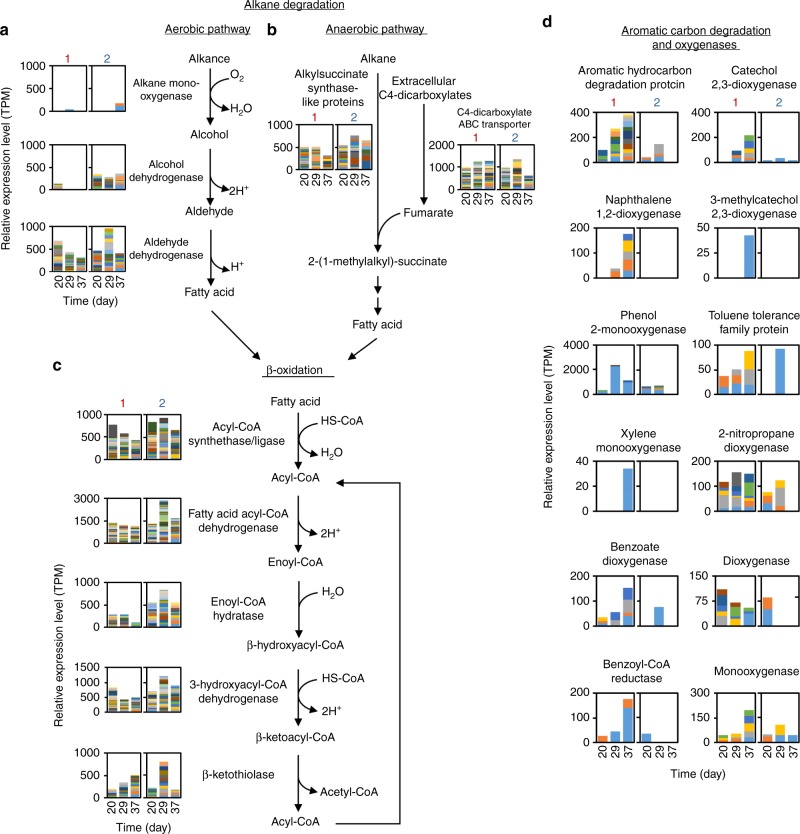
Relative gene expression levels for hydrocarbon and fatty acid degradation pathways. **a** Aerobic alkane degradation. **b** Anaerobic alkane oxidation. **c** Fatty acid degradation (beta-oxidation). **d** Aromatic hydrocarbon-degrading enzymes and oxygenases. For all graphs, left and right panels correspond to the relative expression levels of the genes in reactors 1 and 2, respectively. Color-coded fractions indicate relative expression levels of individual transcript assemblies

The overall trend in gene expression profiles of the two reactors were compared using the correlation plots for relative expression levels of 6371 genes that were commonly expressed in both reactors (Supplementary Fig. 5a–d). The transition of the correlation plots showed that gene expression profiles of the two reactors were initially similar but differed after heavy oil addition, indicating the shifts of active metabolisms in the two reactors. A similar trend was observed in the Pearson correlation coefficients of the microbial community compositions (Supplementary Fig. 5e). In order to extract the key metabolic pathways from the metatranscriptome data, genes that showed increased expression with time (i.e., showed increasing TPM values, see Methods) and that were expressed by a large number of microbe species were listed (Supplementary Data 4). For both reactors, the lists contained genes involved in beta-oxidation, suggesting that alkane, a main component of heavy oil, was degraded via the beta-oxidation pathway after conversion to fatty acid^13,14^. This finding was confirmed by detailed expression analysis of individual genes (Fig. 3c). In general, alkane is first oxidized to fatty acid by alkane monooxygenase under aerobic conditions^13,14^, but in both reactors, genes involved in this process showed low relative expression levels (Fig. 3a). Therefore, we paid attention to an alternative pathway, i.e., anaerobic alkane activation and degradation, in which alkane is added across the double bond of fumarate to form alkyl-substituted succinates via alkylsuccinate synthase^14^. Results for the genes encoding pyruvate formate-lyase and radical-SAM protein, which are homologs of alkylsuccinate synthase and/or share a conserved active domain^14^, are summarized in Fig. 3b as Alkylsuccinate synthase-like proteins; the results suggest that the active pathway was anaerobic degradation rather than aerobic oxidation. In addition, the genes encoding C4-dicarboxylate transporters, which is involved in fumarate uptake^17^, exhibited high expression levels compared with the median values of all transcripts (Fig. 3b, Supplementary Fig. 6), and the relative expression level increased with time in reactor 1 only (Supplementary Data 4). Relative expression levels of genes encoding oxygenases and degradation enzymes for aromatic hydrocarbons were also higher in reactor 1 than in reactor 2 (Fig. 3d, Supplementary Data 5), indicating high degradation activities towards aromatic compounds in reactor 1. Notably, most of the predicted hosts of the expressed genes encoding alkylsuccinate synthase-like proteins and aromatic hydrocarbon degradation enzymes can catalyze nitrate reduction (denitrification) (Supplementary Data 5 and 6)^14^.

**Fig. 4:**
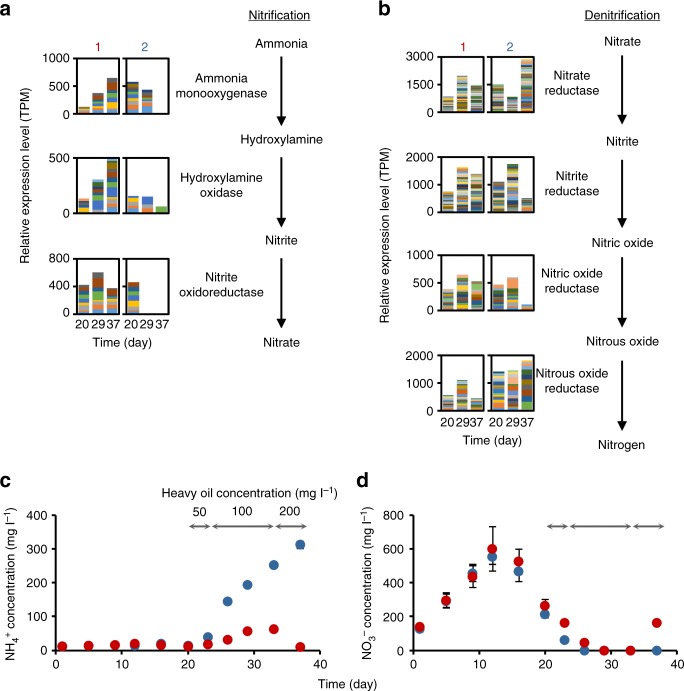
Relative expression levels of genes involved in nitrogen metabolism and concentrations of nitrogen compounds. **a**, **b** Relative expression levels of genes associated with nitrification and denitrification, respectively: left and right panels correspond to levels in reactors 1 and 2, respectively. Color-coded fractions indicate relative expression levels of individual transcript assemblies. **c**, **d** Changes in concentrations of ammonium and nitrate, respectively. Red and blue symbols indicate mean values of two measurements (*n* = 2) for reactors 1 and 2, respectively. Error bars denote ranges

Relative expression levels of denitrification genes were as high as those of tricarboxylic acid cycle genes and aerobic respiration genes in both reactors (Fig. 4, Supplementary Fig. 7a, b), indicating the importance of the anaerobic nitrate respiration even though the sludge was continuously aerated. Because a negligible amount of nitrate is contained in inlet synthetic wastewater, we hypothesized that nitrifiers (ammonia-oxidizing bacteria [AOB] and nitrite-oxidizing bacteria [NOB]) provide nitrate by oxidizing ammonia and nitrite in the reactors^18^. Interestingly, the expression of genes encoding enzymes in the nitrification pathway after heavy-oil addition appeared higher in reactor 1 than in reactor 2 (Fig. 4a; time points 2, 3). The difference in nitrification activities was also supported by the changes in ammonium and nitrate concentrations: in reactor 2, following heavy-oil addition, ammonium accumulated sharply but the nitrate concentration was reduced below the detection limit (Fig. 4c, d); in contrast, in reactor 1, the ammonium concentration was maintained at low levels, and the nitrate concentration increased concomitantly with the decrease in ammonium concentration at day 37 (Fig. 4c, d). Higher activities of AOB in reactor 1 than in reactor 2 were also indicated by the following: (i) relative expression levels of ammonia monooxygenase and hydroxylamine oxidoreductase increased with time in reactor 1 only (Supplementary Data 4; No. 24, 39); (ii) high relative expression levels of nitrification genes after heavy-oil addition were observed in reactor 1 only, as shown by the mapping of mRNA reads onto the *Nitrosomonas* sp. Is79A3 genome (GenBank accession number: NC_015731) (Supplementary Data 7; bold type); (iii) relative expression levels of rRNA genes for *Nitrosospira* and *Nitrosomonas* in reactor 1 were 84- and 6.7-fold those in reactor 2, respectively (Supplementary Fig. 4c, d); and (iv) relative abundances of *Nitrosomonas* sp. in reactor 1 kept increasing after heavy-oil addition, whereas those in reactor 2 decreased after day 27, as revealed by 16S rRNA gene amplicon sequencing (Supplementary Fig. 8). On the other hand, anaerobic alkane degradation has been reported to be catalyzed by sulfate-reducing bacteria as well as denitrifiers^14^. Although sulfate consumption and relative expression levels of genes encoding enzymes in the sulfate reduction pathway were higher in reactor 2 than in reactor 1 (Supplementary Fig. 9), alkylsuccinate synthase-like proteins were apparently not expressed by dissimilatory sulfate reducers. Because the energy available from sulfate reduction is approximately 15-time lower than that from denitrification^19^, sulfate might not induce the effective heavy-oil degradation.

## Discussion

Whether or not aromatic compounds in heavy oil were detoxified by denitrifying bacteria seemed to differentiate the nitrification activities of the respective reactors. Because of their low abundances, nitrifiers could be easily washed out from the sludge microbiome through growth inhibition by the toxic aromatic compounds remaining (Fig. 2g, h, Supplementary Fig. 8). Denitrifiers’ genes encoding degradation enzymes for aromatic hydrocarbons showed high relative expression levels in reactor 1 only (Fig. 3d), presumably removing the toxic compounds to maintain the activity of the susceptible nitrifiers^20^. In turn, nitrifiers helped denitrifiers by providing nitrate. In fact, relative expression levels of AOB-derived rRNA were notably high only in reactor 1 even at the beginning of the heavy-oil addition (Supplementary Fig. 4e, f). This indirect cooperation in the sludge microbiome was vital for sustaining reactor performance. Ammonia monooxygenase and degradation enzymes for aromatic hydrocarbons are generally functional under oxic conditions, whereas enzymes involved in denitrification and anaerobic alkane degradation are activated under anoxic conditions. Dissolved oxygen concentrations in the reactors decreased after the heavy oil addition (Fig. 2c), possibly due to the increase in viscosity of the sludge and the resulting oxygen mass transfer limitation^21,22^. Consequently, both oxic and anoxic conditions existed locally and temporally even in the continuously aerated activated sludge. We proposed that the co-existence of aerobic and anaerobic environments enabled a link between carbon metabolism (alkane and aromatic hydrocarbon degradation) and nitrogen metabolism (nitrification and denitrification), leading to the heavy-oil degradation performances of reactor 1 (Fig. 1).

Here, de novo RNA-seq was proven effective for deciphering functioning metabolisms in a complex sludge microbiome. However, determining which metabolic pathways or reactions cause a phenomenon observed in an ecosystem is challenging, because the phenomenon is usually the sum of diverse biological processes^1–3^. This is especially difficult when the reaction of interest is not identical to, or has little relationship with, the phenomenon, e.g., when these reactions are located far from each other on the metabolic map (Fig. 1). To bypass this drawback, we extracted the vital pathways on the metabolic map by evaluating diversity of the expressed genes (Supplementary Fig. 7d, Table 1). De novo metatranscriptome assembly generated several to dozens of homologous sequences for most assemblies, identifying the higher priority genes that a greater number of microbial species expressed. Given the importance of microbial richness to maintaining ecosystem functionality^23^, the information on the transcript diversity of functioning key genes would be of tremendous benefit. By contrast, RNA-seq studies have so far prioritized genes on the basis of their expression levels alone, because only a few homolog sequences have been available from the limited number of reference genomes or metagenomes. This advantage of de novo RNA-seq can also unmask the ecophysiology of rare microorganisms. The big contribution of minorities, such as AOB and NOB, might not have been identified by a metagenome-based RNA-seq approach.

The results by de novo RNA-seq suggested that the small but important minorities of nitrifiers, AOB and NOB, the relative abundances of which were <0.15% and <0.25%, respectively, governed the heavy-oil degradation activities of the activated sludge bioreactors. Nitrifiers and denitrifiers indirectly cooperated with each other by supplying the respiratory substrate nitrate and by detoxifying heavy-oil components, respectively, thereby helping maintain the reactor performance. Taken together, our de novo RNA-seq strategy deciphers the unexpected linkage between carbon- and nitrogen-metabolisms in the complex microbiome.

## Methods

### Setup and operation of a pilot-scale membrane bioreactor

Schematic configuration of the pilot-scale MBR used in this study, which was the same as our previous studies^22,24,25^, is shown in Supplementary Fig. 10. Operation of the MBR was performed according to previous studies^24,25^. The employed activated sludge was obtained from a municipal wastewater treatment plant (Kinu aqua-station, Ibaraki, Japan). Throughout the experimental period, the bioreactor was constantly fed with a synthetic wastewater stored in a 20-l feed tank at 4 °C. The flow rates of both the input wastewater and the output membrane-filtered treated water were 115 l day^−1^, resulting to a hydraulic retention time of 2 days. The flow of the return sludge from rightmost to leftmost compartments was also set at 115 l day^−1^. No sludge was withdrawn from the reactor, except for periodic sampling. The concentration of the original synthetic wastewater was set at 450 mg-chemical oxygen demand (COD) l^−1^ and the composition was the same as the previous studies^22,24,25^. In addition to synthetic wastewater, heavy oil was supplied to the MBRs at concentrations of 50 mg l^−1^ for days 21–23, 100 mg l^−1^ for days 24–33, and 200 mg l^−1^ for days 34–37 using syringe pump (Pump 11 Elite; Harvard Apparatus, Holliston, MA, USA).

### Procedures for chemical analyses

Fifteen ml of the activated sludge samples were collected and separated into pellet and supernatant by centrifugation. The COD, total organic carbon (TOC) and total nitrogen (TN) values and concentrations of NH_4_^+^, NO_3_^−^, and SO_4_^2−^ in the supernatant and MBR-treated water were analyzed according to the previous reports^22,25^. Heavy oil components were measured as described below: the mixture of 0.25 ml of sludge samples and 2 ml of hexane in a glass test tube was vortexed for 1 min. After separating the two layers, components of the heavy oil in the hexane layer were determined by a Shimadzu GC-mass spectrometer (GCMS-QP 2010, Shimadzu, Kyoto, Japan) that was equipped with a mass spectrometric detector and auto-injector (AOC-20i). For the analysis, 0.25 mm × 25 m by 0.25-μm Quadrex MS capillary column made of 100% dimethyl polysiloxane was used. The GC mass conditions and the temperature program for the GC oven during the analysis were as described previously^22^. Mass data were analyzed at a range of 60.00 *mz*^−1^ to 650.00 *mz*^−1^. All data were represented as the mean values from at least two measurements, except for TOC and TN.

### DNA extraction and PCR amplification

One hundred fifty-six activated sludge samples (i.e., 26-time points × triplicates × two MBRs) periodically obtained from the two MBRs were washed once with a 50 mM sodium phosphate buffer (pH 7.0) and stored at −20 °C as a pellet until use. DNA extraction and purification were performed according to previous studies^24–26^. The V4 region of 16S rRNA genes was amplified using the universal primers 515F and 806R and the purified DNA as a template^4^. Both primers were modified to contain an Illumina adapter region, and the reverse primer contained a 12-bp barcode for multiplex sequencing^27^. The thermal condition of PCR were the same as that in a previous report^24,25^.

### High-throughput sequencing of 16S rRNA gene amplicons

High-throughput sequencing was carried out as described previously^28^. An appropriate amount of the 16S rRNA gene segments and an internal control (PhiX Control V3; Illumina, San Diego, CA, USA) was subjected to paired-end sequencing with a 500-cycles MiSeq reagent kit (Illumina) and a MiSeq sequencer (Illumina). The total number of the sequences obtained from 156 sludge samples (26 time points with triplicate for two reactors) was around 3.51 million, corresponding to an average of 22,473 sequences per library (minimum, 9893; and maximum, 67,099). Removal of PhiX sequences using a Burrows-Wheeler Aligner version 4.0.5 with the database of Greengenes^29,30^, joining the paired-end sequence by ea-utils software package version 1.1.2-301^31^, removal of low-quality (Q < 30) and chimeric sequences by QIIME version 1.7.0 and Mothur version 1.31.2^32,33^, and assembly of the paired-end sequences were carried out according to a previous report^34^. Microbial diversity of each sequence library was characterized by calculating α-diversity indices (i.e., Chao1, Shannon, and Simpson reciprocal) and the weighted UniFrac distances for PCoA using the QIIME software. The closest relative of the OTU was determined by aligning 16S rRNA sequences using Blast search (https://blast.ncbi.nlm.nih.gov/Blast.cgi) with NCBI nucleotide sequence database.

### RNA extraction and sequencing

Two-milliliter samples of activated sludge were collected on days 20, 29, and 37 from the two reactors (i.e., total 6 samples) and immediately centrifuged (15,300 × *g*, 5 min, 4 °C), and the resulting pellets were stored at −80 °C until use. RNA was extracted from the pellet by the direct lysis protocol, as described above. The DNA-contaminated extract was digested with DNase (RQ1 RNase-Free DNase, Promega, Fitchburg, WI, USA) and purified by using an RNeasy Mini Kit (QIAGEN, Venlo, Netherlands). The resultant total RNA was treated by using a RiboMinus Transcriptome Isolation Kit for bacteria (Thermo Fisher Scientific) to reduce rRNA amounts, and purified by using an AMPure XP kit. Double-stranded cDNA libraries were prepared from the rRNA-depleted RNA samples by using a NextUltraRNA library prep kit (NEB). The target size of cDNA (200–300 bp) was purified by using an AMPure XP kit followed by agarose-gel electrophoresis, as described above. The size distribution and concentration of the purified cDNA samples were determined by using a Bioanalyzer (Agilent 2100; Agilent) and the Quant-iT PicoGreen dsDNA reagent and kit, respectively. An eight picomolar cDNA sample was subjected to paired-end sequencing with a 300-cycle MiSeq reagent kit and a MiSeq sequencer. RNA sequencing using the MiSeq platform provided 31,882,097 reads in total, corresponding to an average of 5,313,683 sequences per library (minimum, 2,604,746; maximum, 7,979,418; Supplementary Table 1).

### Sequence analysis of transcripts

Paired-end Illumina reads from each of six samples were checked for quality with FastQC (a quality control tool for high-throughput sequence data, available online at: http://www.bioinformatics.babraham.ac.uk/projects/fastqc). To ensure high sequence quality, the remaining sequencing adaptors and the reads with a cutoff Phred score of 15 (for leading and tailing sequences, Phred score of >20) and a length of less than 100 bp were removed by the program Trimmomatic v0.30 using Illumina TruSeq3 adapter sequences for adapter clipping^35^. After the removal of low-quality sequences, 15,338,269 reads survived in total, corresponding to an average of 2,556,378 reads per sample (minimum, 1,460,332; maximum, 3,605,484; Supplementary Table 1). The remaining paired reads were reconstructed into long transcripts by using the de novo assembly program Trinity 2.1.1^9^, generating 23,709 and 20,677 assemblies for reactors 1 and 2, respectively (Supplementary Table 1). Using constructed assemblies as references, paired-end RNA reads were mapped with the Bowtie 2 program^36^. An average of 74.1% of RNA sequences were mapped onto the constructed assemblies (minimum, 69%; maximum, 80%; Supplementary Table 1). After conversion of the output BAM files to BED files with the bamtobed program in BEDTools ver. 2.14.3^37^, relative expression levels of transcripts were evaluated by calculating TPM (Transcripts Per kilobase Million) values by using in-house scripts; relative gene expression levels were calculated by first dividing the mapped read counts by the length of each gene in kilobases, giving reads per kilobase (RPK); then, all the RPK values in a sample were counted up and divided by 1,000,000 (per million scaling factor), giving TPM value. For evaluating the similarity of gene expression profiles between the two reactors, the correlation plots for relative expression levels of 6,630 genes that were commonly expressed in both reactors were used. The commonly expressed genes were extracted by bidirectional homology search using Blastn program, in which the assembly sets exhibiting the highest homology with each other (bi-direction best hits) were selected. Relative expression levels of three reference genes commonly used as housekeeping genes in transcriptome analysis, and those for genes encoding tricarboxylic acid cycle enzymes, which are conserved across various microorganisms and are known to actively function in aerobic environments, were summarized to confirm the validity of the evaluation on the basis of TPM values (Supplementary Figs. 6 and 7). Furthermore, frequency distribution plots of relative expression levels of all genes were prepared to confirm the overall profiles of the expression levels and to estimate the criteria for highly expressed genes. For generating the frequency plots, first the relative expression levels of assemblies which are assigned to the same functional gene were summed up (e.g., when expression levels of assemblies 1, 2, and 3, all of which are assigned to gene A, were 10, 20 and 30, respectively, the total expression level of gene A is 60); then, the frequency of the total expression levels and median value were calculated.

### Assignment of transcript functions

Functions of the transcripts were predicted by a homology search using four analytical programs and eight gene databases (Supplementary Table 1). Ribosomal RNA sequences were assigned by riboPicker 0.4.3 with reference to SILVA rRNA database version 123^38,39^. Sequences for 5S rRNA, and tmRNA were assigned by Fasta36 aligner with reference to 5S rRNAdb and tmRDB^40–42^, respectively, when the e-value of the output was less than 1e−10. For Fasta36 analyses, 5S rRNAdb and tmRDB were prepared in FASTA format by using in-house scripts. Transfer RNA sequences were assigned by tRNAscan-SE-1.3.1 with the general tRNA model option^43^. The functions of coding sequences (CDSs) were predicted by a homology search using Blastx (ncbi-blast-2.2.29+) with reference to the NCBI reference sequence database (RefSeq, release 64)^44,45^, EggNOG database v4.0, or GO database^46,47^, or by using PfamScan with reference to the Pfam database after converting nucleic acid sequences to amino acid sequences by using TransDecoder (available online at http://transdecoder.sourceforge.net/)^48^. Only the functions with high scores (alignment length, >50 amino acids; homology, ≥25%) were assigned to CDSs^49^. Here, the gene annotation by RefSeq was used, because the most gene functions were assigned by using RefSeq from among the four databases (Supplementary Table 1). The proportions of CDSs and rRNA, tRNA, and tmRNA sequences aligned to assemblies were estimated at approximately 85%, 11%, 0.36%, and 1%, respectively. However, because rRNA, tRNA and tmRNA accounted for approximately 91%, 0.16%, and 1.3%, respectively, of the total expression level, TPM values for the CDS analysis were recalculated without the rRNA, tRNA, or tmRNA assemblies^50^.

### Ribosomal RNA expression analysis

To investigate which microbial taxa contributed to the heavy-oil degradation, relative expression levels of ribosomal RNA were calculated. Ribosomal RNA sequences discriminated by using riboPicker were phylogenetically classified by using Fasta36 with reference to the SILVA rRNA database (both large and small subunit) with a cutoff e-value of 1e–10. TPM values of each taxonomic group (at class and genus levels) were assigned with in-house scripts.

### Extraction of increasingly expressed genes

To explore the overall trends in gene expression profiles, genes that were highly expressed after heavy-oil addition were investigated. First, genes with relative expression levels that increased with time (i.e., genes with TPM values at day 20 < day 29 < day 37) were extracted; accordingly, 2078 and 1515 genes were identified in reactor 1 and 2, respectively. Then, the number of gene homologs, which were discriminated as different transcript assemblies by using Trinity, were counted; we considered this number to be correlated with the importance of the gene, because genes that play an important role in the environment would be expected to be expressed by various organisms. For this analysis, gene functions were assigned with various criteria (i) alignment length, >50 amino acids; homology, ≥25%; (ii) alignment length, >50 amino acids; homology, ≥50%, (iii) homology, ≥50%, (iv) bit score, ≥40; homology, ≥50%, and (v) bit score, ≥40. We confirmed that all analyses using the various annotation criteria showed almost the same gene-expression trends, although several genes, such as those encoding nitrate reductase or C4-dicarboxylate transporter, were detected by criterion (i) only. Therefore, the results obtained using criteria (i) (cutoff value ≥5 homologs) are shown in the manuscript (Supplementary Data 4).

### Evaluation of transcripts by integrating expression levels

For several metabolic pathways that we focused on (e.g., alkane degradation), stacked bar graphs of relative expression levels were generated by integrating the TPM values (Supplementary Fig. 7d). Because annotations of genes are diversified (i.e., various names are assigned to the same functional gene), we manually summarized annotations into one representative annotation according to the KEGG database (http://www.genome.jp/kegg/genes.html). In addition, although the subunit composition of isozymes often varies among host microorganisms, we did not discriminate subunit information, and this may have led to some bias in the relative expression levels. Furthermore, when an assembly showed a notably high TPM value (e.g., >500), the annotation of the assembly was analyzed again by using Blastn or Blastx with reference to non-redundant NCBI databases (http://www.ncbi.nlm.nih.gov/RefSeq/), to avoid misannotation.

### Mapping of RNA sequences onto the *Nitrosomonas* genome

Because the expressed ammonia monooxygenase genes shared high homology with the homologs of *Nitrosomonas* sp. Is79A3, *Nitrosomonas* sp. AL212, or *Nitrosomonas eutropha*, the RNA sequences (after quality control by Trimmomatic) were mapped onto the publicly available *Nitrosomonas* sp. Is79A3 genome by using the Bowtie 2 program. Calculation of TPM values was performed as described above.

### Reporting summary

Further information on research design is available in the Nature Research Reporting Summary linked to this article.

## Supplementary information

Reporting Summary

Supplementary information

Description of Additional Supplementary Files

Supplementary Data 1

Supplementary Data 2

Supplementary Data 3

Supplementary Data 4

Supplementary Data 5

Supplementary Data 6

Supplementary Data 7

## Data Availability

The amplicon sequencing data and the metatranscriptome data of this study have been deposited in the DDBJ Sequence Read Archive under accession code DRA006299 and DRA006303, respectively. All source data underlying the graphs presented in the main figures are available as Supplementary Data 1–3.
