# Supplementary material for: Transcriptome analysis of activated sludge microbiomes reveals an unexpected role of minority nitrifiers in carbon metabolism
Source: Commun Biol. 2019 May 13;2:179. doi: 10.1038/s42003-019-0418-2 (PMC6513846; doi:10.1038/s42003-019-0418-2)
Supplement: Supplementary file 1 — Reporting Summary [file 42003_2019_418_MOESM1_ESM.pdf]

nature research

Corresponding author(s): Tomoyuki HORI

Last updated by author(s): Mar 15, 2019

Reporting Summary

Nature Research wishes to improve the reproducibility of the work that we publish. This form provides structure for consistency and transparency in reporting. For further information on Nature Research policies, see Authors & Referees and the Editorial Policy Checklist.

Statistics

For all statistical analyses, confirm that the following items are present in the figure legend, table legend, main text, or Methods section.

n/a	Confirmed	
☐	☒	The exact sample size (*n*) for each experimental group/condition, given as a discrete number and unit of measurement
☐	☒	A statement on whether measurements were taken from distinct samples or whether the same sample was measured repeatedly
☐	☒	The statistical test(s) used AND whether they are one- or two-sided<br>*Only common tests should be described solely by name; describe more complex techniques in the Methods section.*
☒	☐	A description of all covariates tested
☐	☒	A description of any assumptions or corrections, such as tests of normality and adjustment for multiple comparisons
☐	☒	A full description of the statistical parameters including central tendency (e.g. means) or other basic estimates (e.g. regression coefficient) AND variation (e.g. standard deviation) or associated estimates of uncertainty (e.g. confidence intervals)
☒	☐	For null hypothesis testing, the test statistic (e.g. *F*, *t*, *r*) with confidence intervals, effect sizes, degrees of freedom and *P* value noted<br>*Give P values as exact values whenever suitable.*
☒	☐	For Bayesian analysis, information on the choice of priors and Markov chain Monte Carlo settings
☒	☐	For hierarchical and complex designs, identification of the appropriate level for tests and full reporting of outcomes
☐	☒	Estimates of effect sizes (e.g. Cohen's *d*, Pearson's *r*), indicating how they were calculated

Our web collection on statistics for biologists contains articles on many of the points above.

Software and code

Policy information about availability of computer code

Data collection

For all data collection, instrument data was obtained using the manufacturer recommended software.

Data analysis

For 16S rRNA amplicon sequence analysis: PhiX sequences were removed from raw reads using a Burrows-Wheeler Aligner version 4.0.5 with the database of Greengenes 13_8; The paired-end sequences were joined by ea-utils software package version 1.1.2-301; Low-quality (Q<30) and chimeric sequences were removal by QIIME version 1.7.0 and Mothur version 1.31.2; The paired-end sequences were assembled using QIIME 1.7.0; Phylogenetic characterization of the sequences and calculation of the α-diversity indices (e.g., Chao1, Shannon, and Simpson reciprocal) and the weighted unifrac distances (for PCoA analysis) were performed using QIIME 1.7.0; The closest relative of the operational taxonomic units was determined based on the comparison of their 16S rRNA sequences with those in the DDBJ nucleotide sequence database during Blast searches (https://blast.ncbi.nlm.nih.gov/Blast.cgi).
For metatranscriptome analysis: Paired-end reads were checked for quality with FastQC (http://www.bioinformatics.babraham.ac.uk/projects/fastqc); the remaining sequencing adaptors and the reads with a cutoff Phred score of 15 (leading and tailing sequences, Phred score of >20) and a length of less than 100 bp were removed by the program Trimmomatic v0.30 using Illumina TruSeq3 adapter sequences for adapter clipping; Reconstruction of the paired reads into long transcripts were performed using the de novo assembly program Trinity 2.1.1; The paired-end reads were mapped using the constructed assemblies as references, with the Bowtie2 program; BAM files were converted to BED files with the bamtobed program in BEDTools ver. 2.14.3; Ribosomal RNA sequences were assigned by riboPicker 0.4.3 with reference to SILVA rRNA database version 123; Sequences for 5S rRNA and tmRNA were assigned by Fasta36 aligner with reference to 5S rRNAdb and tmRDB, respectively; Transfer RNA sequences were assigned by tRNAscan-SE-1.3.1 with the general tRNA model option; The functions of coding sequences were predicted by a homology search using Blastx (ncbi-blast-2.2.29+) with reference to the NCBI reference sequence database (RefSeq, release 64), EggNOG database v4.0, or GO database, or by using PfamScan with reference to the Pfam database after converting nucleic acid sequences to amino acid sequences by using TransDecoder (http://transdecoder.sourceforge.net/); Mapping of the paired-end reads onto the Nitrosomonas sp. Is79A3 genome was performed using the Bowtie2 program.

For manuscripts utilizing custom algorithms or software that are central to the research but not yet described in published literature, software must be made available to editors/reviewers. We strongly encourage code deposition in a community repository (e.g. GitHub). See the Nature Research guidelines for submitting code & software for further information.

Data

Policy information about availability of data

All manuscripts must include a data availability statement. This statement should provide the following information, where applicable:

- Accession codes, unique identifiers, or web links for publicly available datasets
- A list of figures that have associated raw data
- A description of any restrictions on data availability

The data availability statement has been included in the manuscript. The amplicon sequencing data and the metatranscriptome data of this study have been deposited in the DDBJ Sequence Read Archive under accession code DRA006299 and DRA006303, respectively.

Field-specific reporting

Please select the one below that is the best fit for your research. If you are not sure, read the appropriate sections before making your selection.

☒ Life sciences ☐ Behavioural & social sciences ☐ Ecological, evolutionary & environmental sciences

For a reference copy of the document with all sections, see nature.com/documents/nr-reporting-summary-flat.pdf

Life sciences study design

All studies must disclose on these points even when the disclosure is negative.

Sample size	For operation of pilot-scale bioreactors, in general, experiments without the replicates of bioreactors are acceptable. In this study, we operated two pilot-scale bioreactors without respective replicates.<br>For metatranscriptome analysis of environmental samples, in general, experiments without replicate are acceptable. We prepared single metatranscriptome library at three time points for individual bioreactors.<br>For 16S rRNA gene amplicon sequencing and chemical analyses, generally accepted sample numbers (n=1~3) were chosen.
Data exclusions	No data were excluded from the study.
Replication	For operation of pilot-scale bioreactors, we confirmed reproducibility of the reactor performances based on those operated in previously reported studies (Ref. 22, 25, 26).<br>For metatranscriptome analysis, we confirmed reproducibility by the correlation plots for expression levels of 6,630 genes that were commonly expressed in the both reactors (Supplementary Fig. 5). Further, expression levels of three reference genes, which are commonly used as housekeeping genes in transcriptome analysis, were compared at three time points in the two bioreactors (Supplementary Fig. 7).<br>For 16S rRNA amplicon sequencing and chemical analyses, reproducibility was confirmed by comparison among the replicates as well as among the time course data (26 time points for measurements of TOC, TMP, DO, MLSS, and sequencing analysis of 16S rRNA gene; 11 time points for measurements of alkanes, PAHs, ammonium, nitrate, COD and sulfate)
Randomization	Activated sludge samples were periodically obtained from two bioreactors. Sample groups divided according to reactor identity and time series.
Blinding	There are no blinding data and aspects included in this manuscript.

Reporting for specific materials, systems and methods

We require information from authors about some types of materials, experimental systems and methods used in many studies. Here, indicate whether each material, system or method listed is relevant to your study. If you are not sure if a list item applies to your research, read the appropriate section before selecting a response.

Materials & experimental systems

n/a	Involved in the study
☒	☐ Antibodies
☒	☐ Eukaryotic cell lines
☒	☐ Palaeontology
☒	☐ Animals and other organisms
☒	☐ Human research participants
☒	☐ Clinical data

Methods

n/a	Involved in the study
☒	☐ ChIP-seq
☒	☐ Flow cytometry
☒	☐ MRI-based neuroimaging
