# Supplementary material for: Transcriptome analysis of activated sludge microbiomes reveals an unexpected role of minority nitrifiers in carbon metabolism
Source: Commun Biol. 2019 May 13;2:179. doi: 10.1038/s42003-019-0418-2 (PMC6513846; doi:10.1038/s42003-019-0418-2)
Supplement: Supplementary file 2 — Supplementary information [file 42003_2019_418_MOESM2_ESM.pdf]

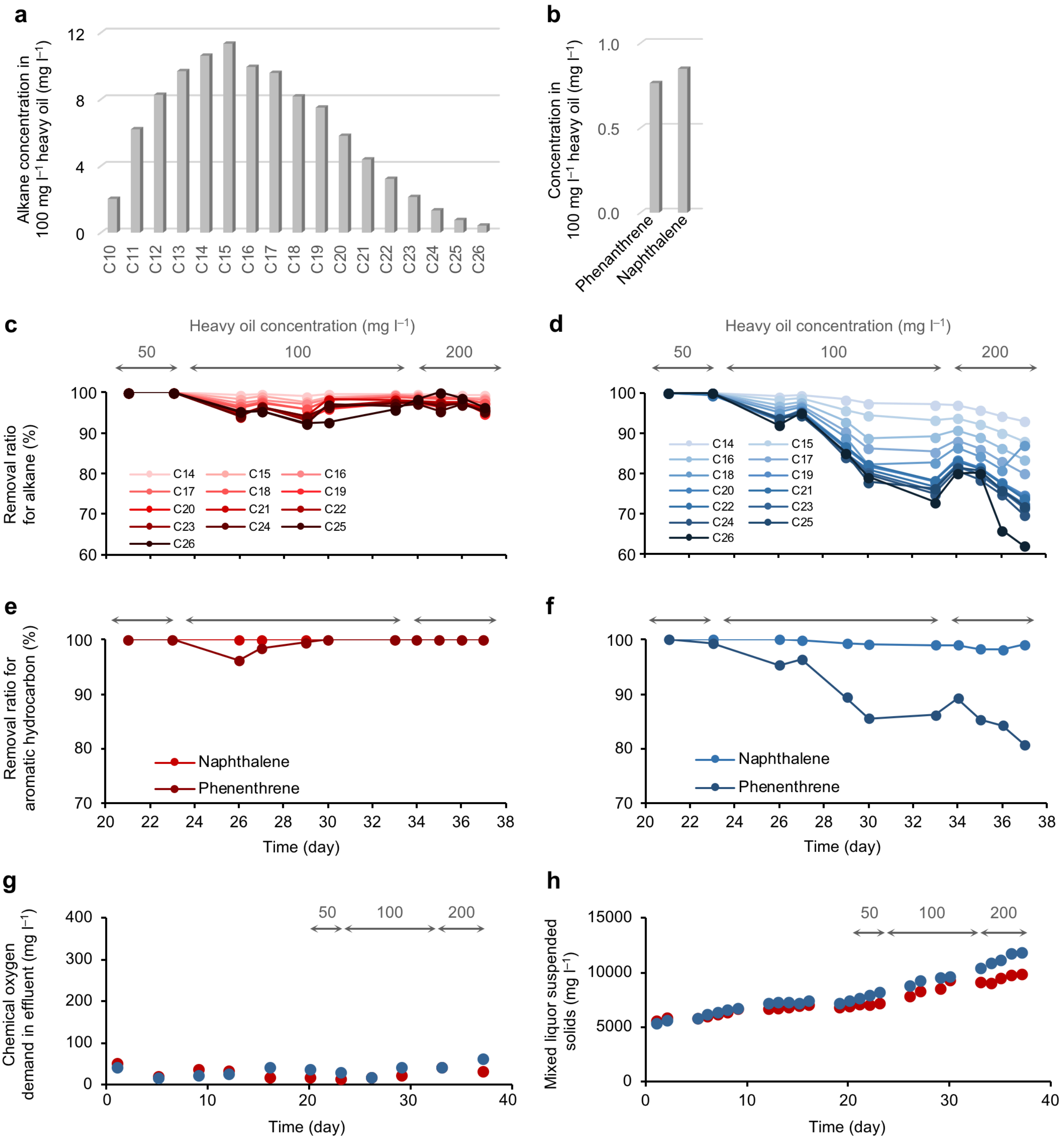

Supplementary Figure 1. Hydrocarbon concentration and physicochemical parameters of the reactors. a, Alkane and **b,** polycyclic aromatic hydrocarbon (phenanthrene and naphthalene) concentrations in initial heavy oil (100 mg l^{-1}) were analyzed by using a Shimadzu GC-mass spectrometer (GCMS-QP 2010) equipped with a mass spectrometric detector and auto-injector (AOC-20i). Alkanes are shown in order of increasing molecular weight (C10 to C26; left panel). **c–f,** Removal ratios of alkanes (C14 to C25) and polycyclic aromatic hydrocarbons were calculated on the basis of gas chromatography – mass spectrometry measurements. **g,** Mixed liquor suspended solids were measured daily. **b,** Chemical oxygen demand (COD) in effluent was analyzed by using a

COD analyzer (DR2800 and DRB200). COD and hydrocarbon concentrations are mean values of duplicate measurements. Red and blue symbols indicate reactors 1 and 2, respectively.

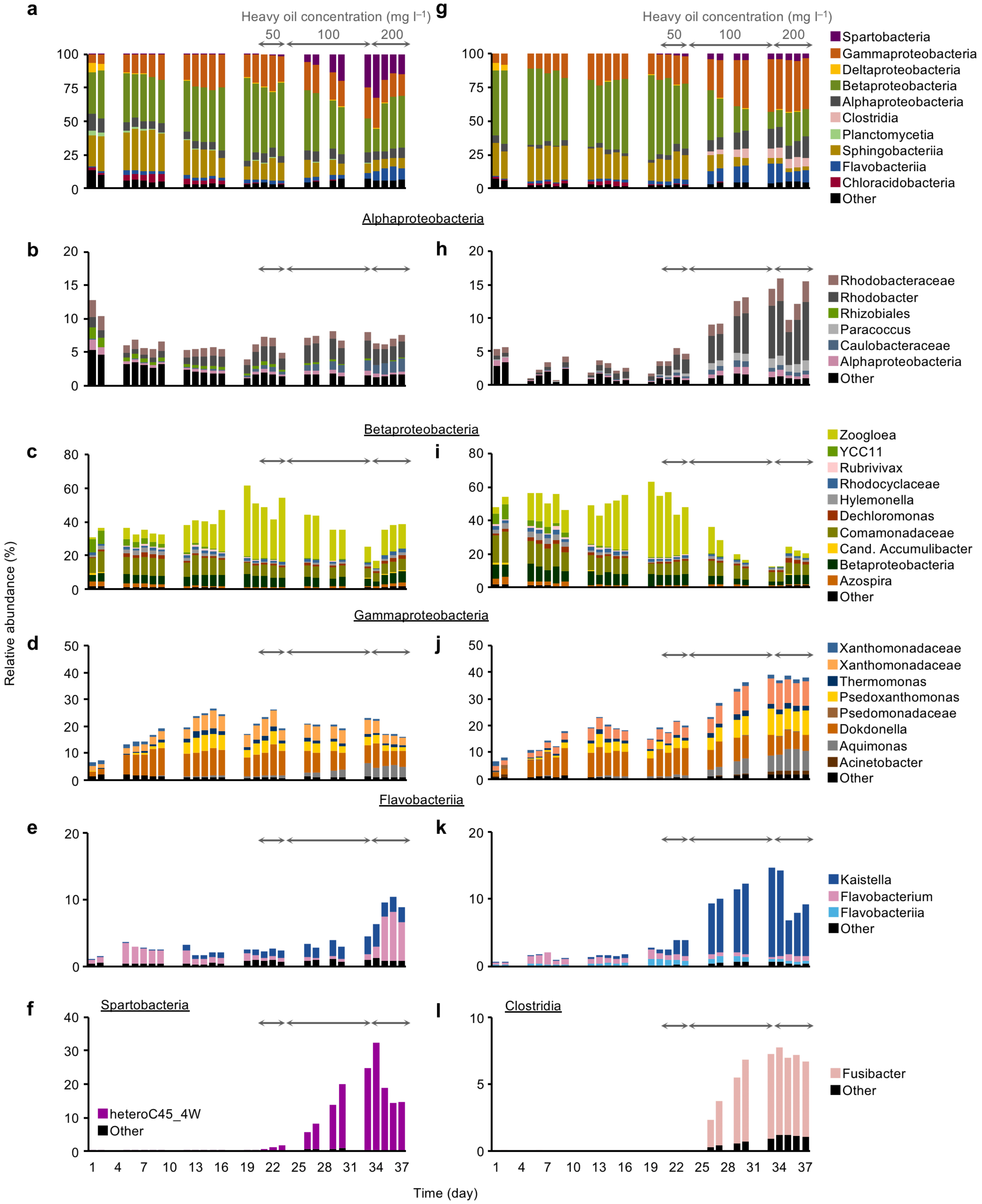
a
g
Heavy oil concentration (mg l−1)
50
100
200
Spartobacteria
Gammaproteobacteria
Deltaproteobacteria
Betaproteobacteria
Alphaproteobacteria
Clostridia
Planctomycetia
Sphingobacteriia
Flavobacteriia
Chloracidobacteria
Other
Alphaproteobacteria
b
h
Rhodobacteraceae
Rhodobacter
Rhizobiales
Paracoccus
Caulobacteraceae
Alphaproteobacteria
Other
Betaproteobacteria
c
i
Zoogloea
YCC11
Rubrivivax
Rhodocyclaceae
Hylemonella
Dechloromonas
Comamonadaceae
Cand. Accumulibacter
Betaproteobacteria
Azospira
Other
Gammaproteobacteria
d
j
Xanthomonadaceae
Xanthomonadaceae
Thermomonas
Psedoxanthomonas
Psedomonadaceae
Dokdonella
Aquimonas
Acinetobacter
Other
Flavobacteriia
e
k
Kaistella
Flavobacterium
Flavobacteriia
Other
f
Spartobacteria
heteroC45_4W
Other
l
Clostridia
Fusibacter
Other
Relative abundance (%)
Time (day)

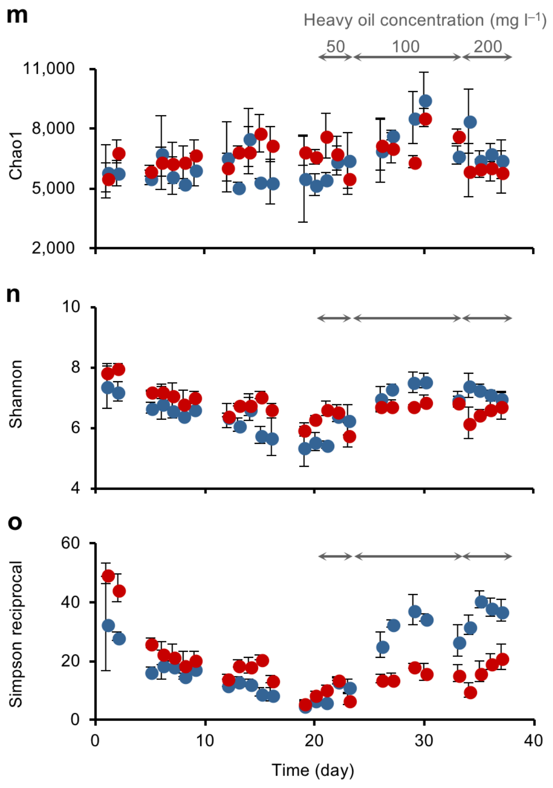

Supplementary Figure 2. 16S rRNA gene analysis of bacterial community structure and the alpha diversity indices. Microbial community structure in activated sludge samples was analyzed by high-throughput sequencing of 16S rRNA gene amplicons prepared from the sludge DNA. Relative distributions of the sequences at the class level (**a,** reactor 1; **g,** reactor 2) and genus level are shown (**b, h,** Alphaproteobacteria; **c, i,** Betaproteobacteria; **d, j,** Gammaproteobacteria; **e, k,** Flavobacteria; **f,** Spartobacteria; **l,** Clostridia). Classes and genera with a relative abundance of more than 3% and 1%, respectively, are displayed individually. Relative abundance was calculated as the mean of triplicates. Taxonomic categories were identified by using QIIME. Left and right panels correspond to the relative abundances in reactors 1 and 2, respectively. Three alpha diversity indices—**m,** Chao1; **n,** Shannon; and **o,** Simpson reciprocal—were calculated on the basis of equal amounts of sequences (9,702) sub-sampled 10 times from original libraries. For each index, higher values represent more diverse microbial communities. Red and blue symbols indicate mean values (n = 3) for reactors 1 and 2, respectively. Error bars denote standard deviations.

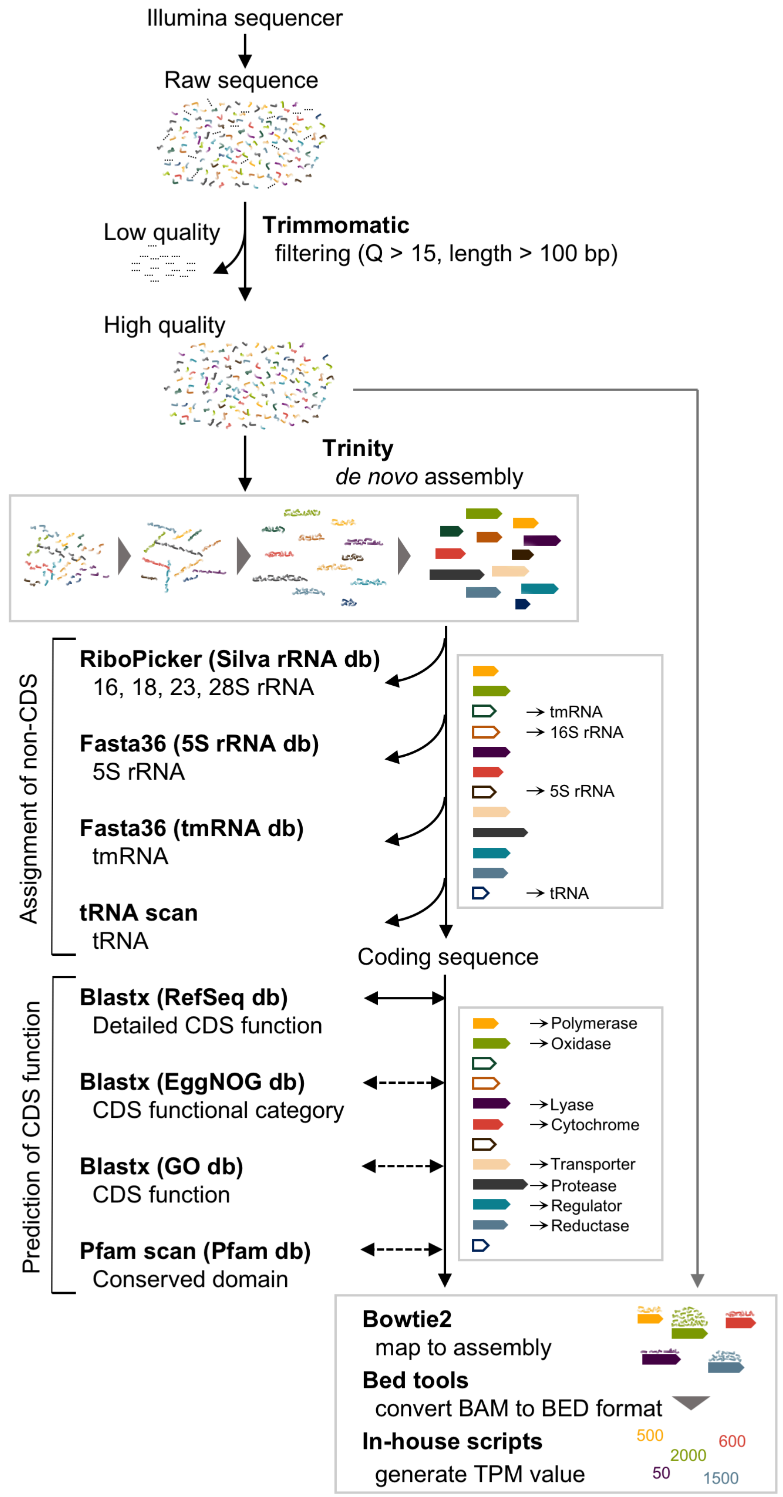

Supplementary Figure 3. Scheme of sequence data analysis. Low-quality sequences were removed from the raw reads. The cDNA sequences were *de novo* assembled into long transcripts by using Trinity[9]. Ribosomal RNA (16, 18, 23, and 28S), 5S rRNA, tmRNA, and tRNA sequences were removed, and then gene functions for coding sequences (CDSs) were assigned by conducting Blastx

searches of the RefSeq database[44,45]. Gene expression levels were calculated as TPM values (relative abundance of transcripts) by using Bowtie2 and Bed tools[36,37].

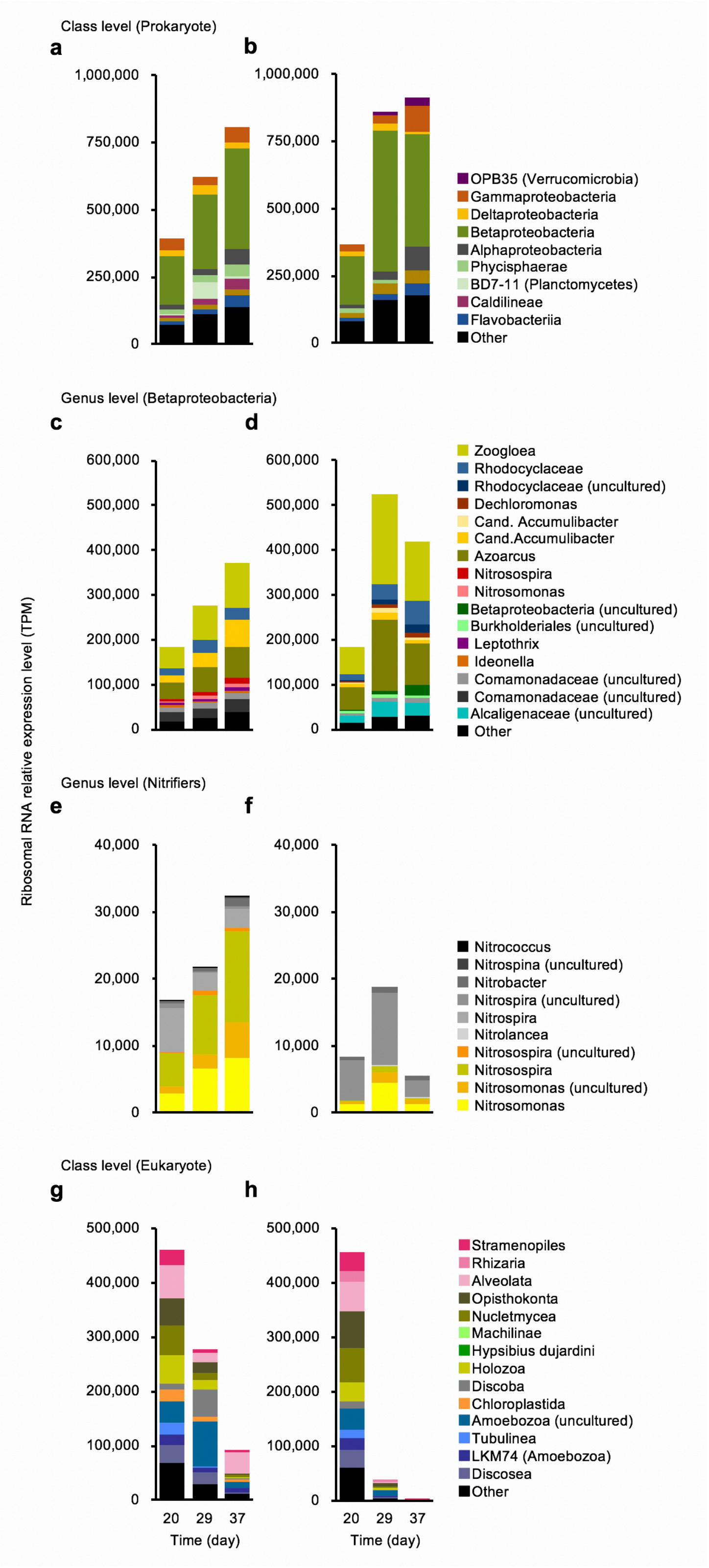

Supplementary Figure 4. Prokaryotic and eukaryotic microbial community structures based on 16S, 18S, 23S and 28S rRNA gene expression. Ribosomal RNA assemblies were discriminated by using riboPicker and phylogenetically classified by using Fasta36 with reference to the SILVA rRNA database at the level of class (**a, b,** Prokaryote; **g, h,** Eukaryote) or genus (**c, d,** genera in

Betaproteobacteria; **e, f,** genera in Nitrifier)[38–40]. The relative expression levels (TPM) of each assembly was calculated by using in-house scripts. Classes and genera that showed relative abundances of more than 3% and 1%, respectively, are displayed individually. Larger taxonomic categories indicate ones unclassified into lower categories. Left and right panels correspond to the relative expression levels of rRNA in reactors 1 and 2, respectively.

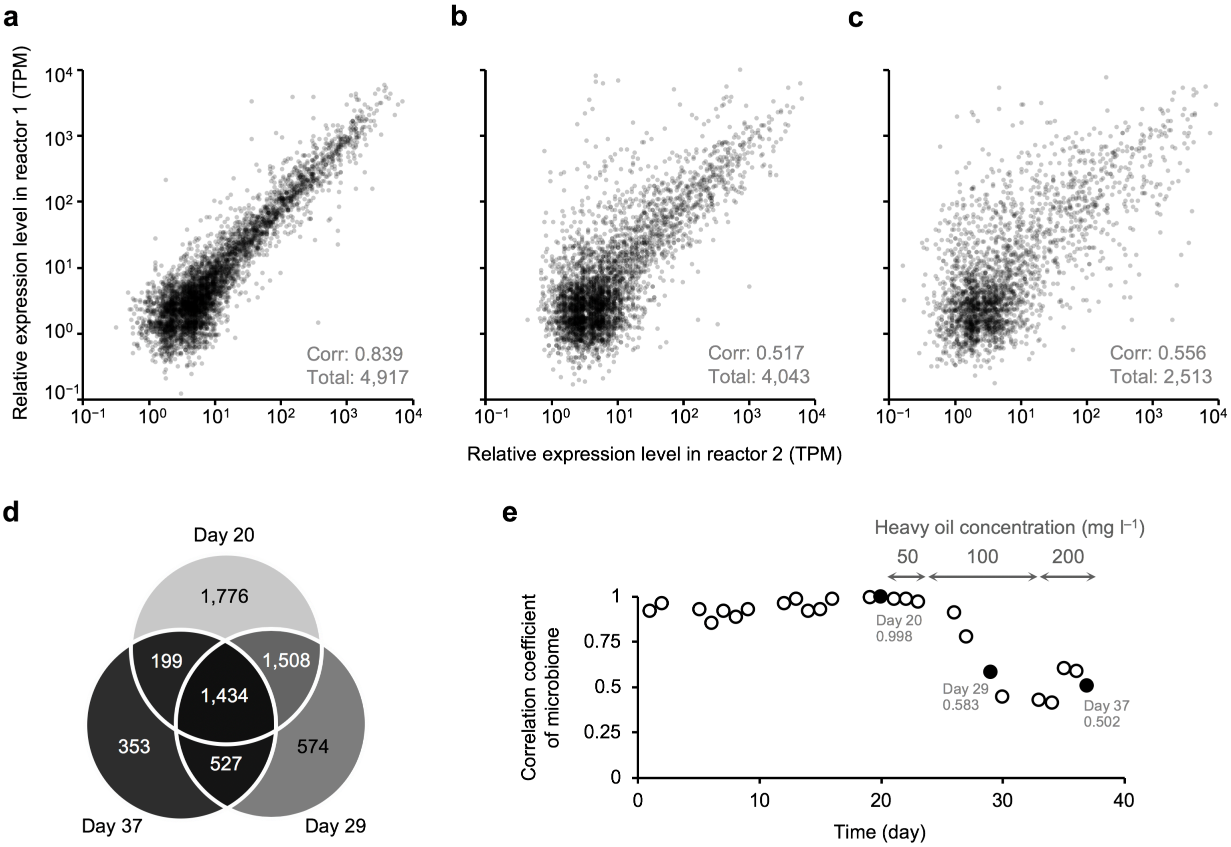

Supplementary Figure 5. Correlation plots of transcripts and transition of correlation coefficient of microbiome. Correlation plots for gene expression levels were generated based on the relative expression levels of 6,371 genes that were commonly expressed in the both reactors. Pearson correlation coefficients and gene number used in the plots were 0.839 and 4,917 for day 20 (**a**); 0.517 and 4,043 for day 29 (**b**); and 0.556 and 2,513 for day 37 (**c**); respectively. Overlap of the 6,371 total expressed genes in the three correlation plots is shown in Venn diagram (**d**). Pearson correlation coefficients of the microbial community structures of the two reactors were calculated using DNA-based relative abundances of 1,431 OTUs (**e**).

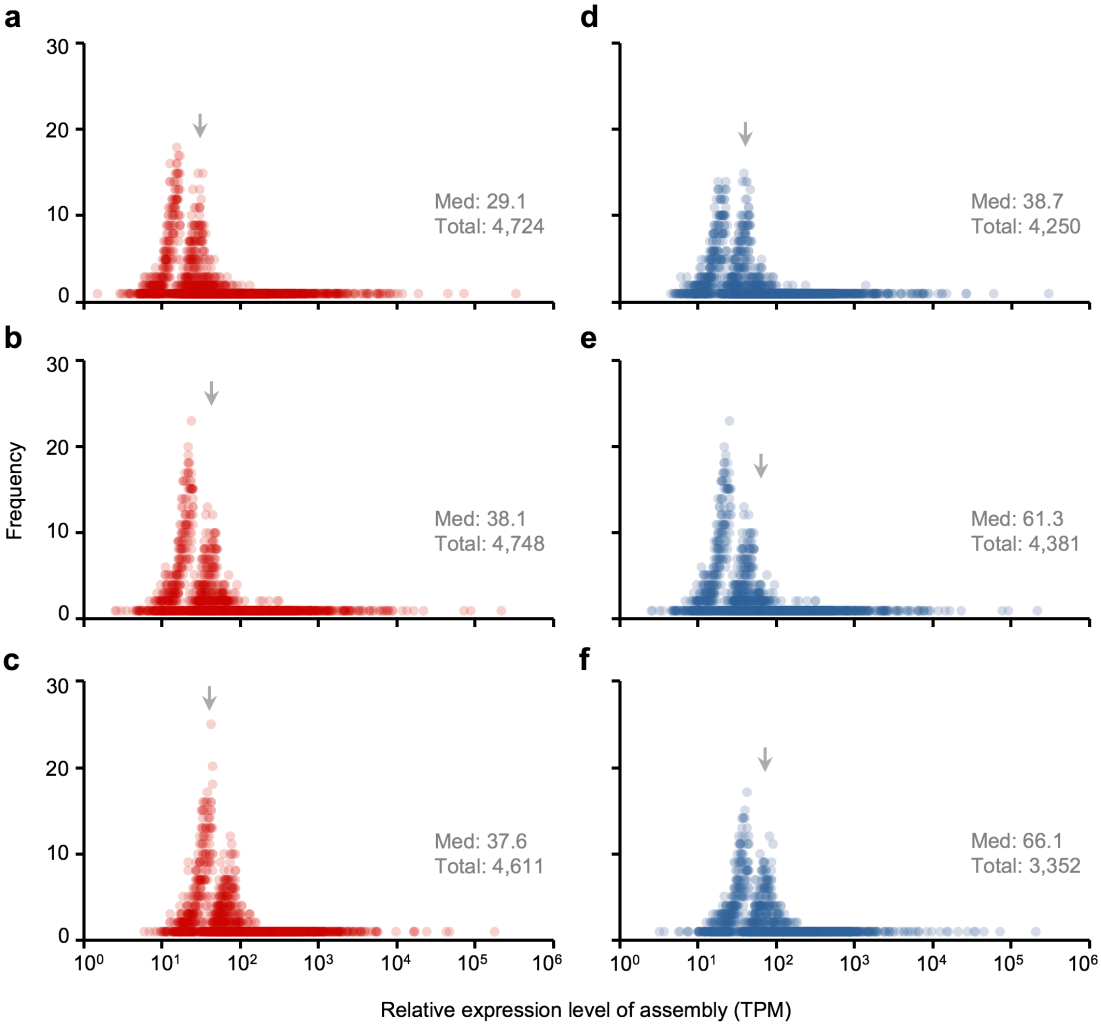

Supplementary Figure 6. Frequency distribution plots of the relative expression levels of all the genes detected. Frequency distribution plots of the relative expression levels of genes detected in reactor 1 (**a,** day 20; **b,** day 29; and **c,** day37) and reactor 2 (**d,** day 20; **e,** day 29; and **f,** day37). Arrows indicate the position of median values. The total numbers of genes used for generating frequency distribution plots are denoted.

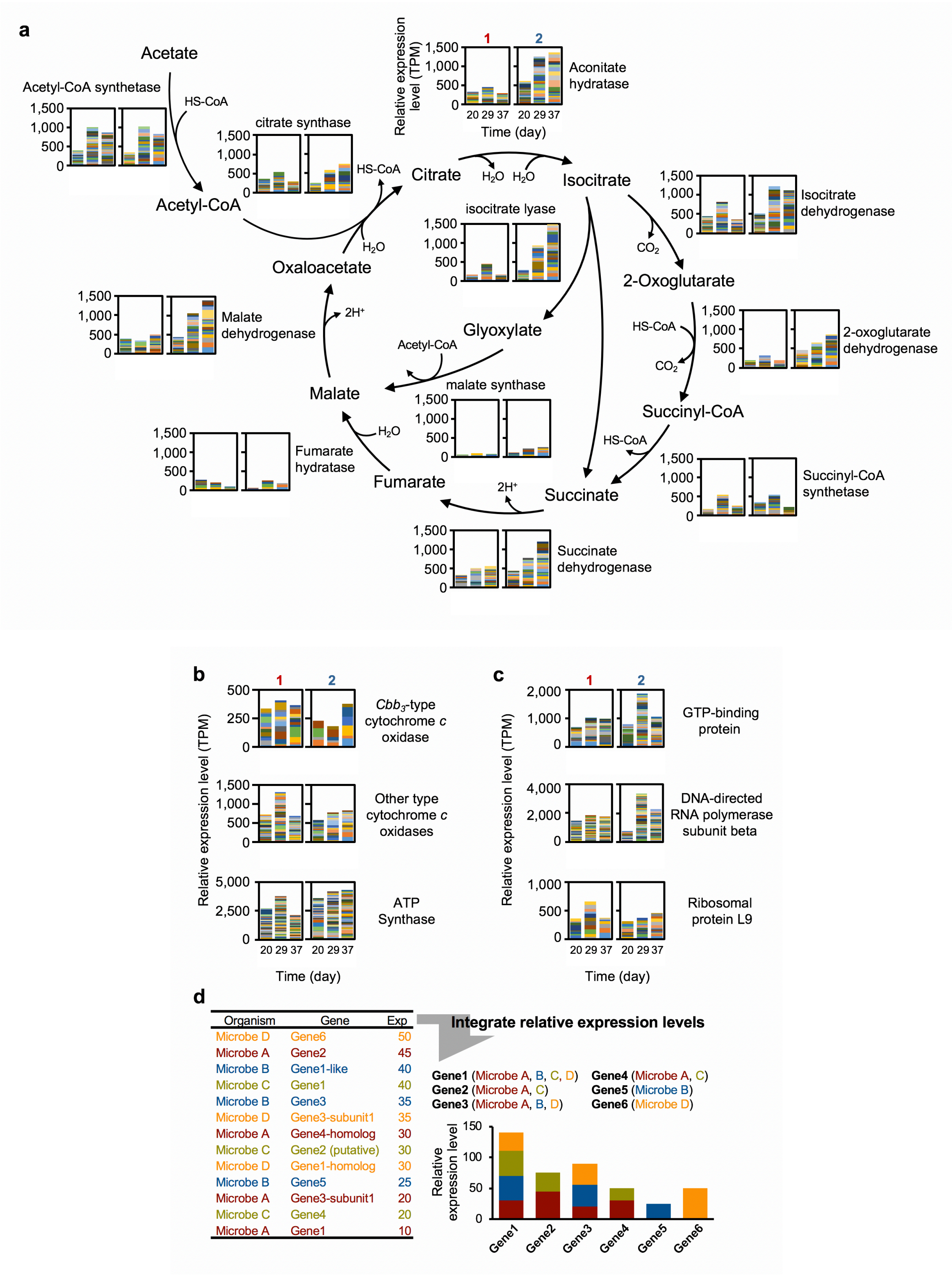

Supplementary Figure 7. Relative expression levels of major metabolic genes and reference genes and evaluation strategy for transcripts by integrating relative expression levels of homologues. Relative expression levels of genes for **a,** tricarboxylic acid (TCA) cycle enzymes and **b,** cytochrome *c* oxidases and ATP synthase are shown. Because the TCA cycle and aerobic respiration is key metabolic pathways for heterotrophs, various organisms possess homologous genes encoding enzymes in this cycle, and such genes are generally highly expressed. *Cbb*$_3$-type

cytochrome oxidase has high affinity for oxygen. **c,** Relative expression levels of three reference genes commonly used as housekeeping genes in transcriptome analysis were analyzed. Left and right panels correspond to the relative expression levels in reactors 1 and 2, respectively. The color-coded fractions indicate relative expression levels of individual transcript assemblies. **d,** Individual genes were evaluated by integrating the relative expression levels of their homologues. Evaluating each homologue by relative expression level alone could lead to misunderstanding of the priority of metabolic pathways.

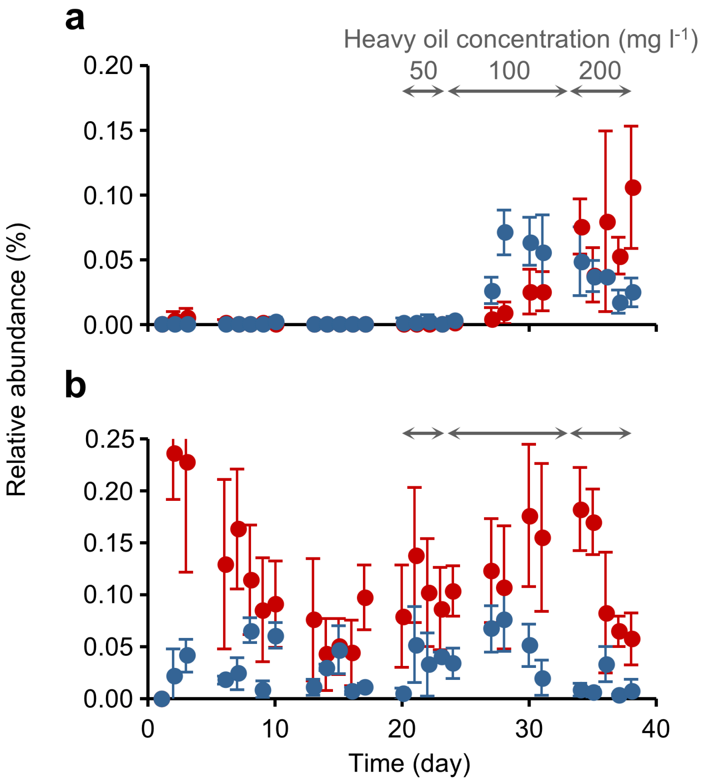

Supplementary Figure 8. Changes in relative abundance of nitrifiers. Changes in relative abundance of **a,** ammonia oxidizing bacteria (AOB; *Nitrosomonas*) and **b,** nitrite oxidizing bacteria (NOB; *Nitrospira*) were calculated by using 16S rRNA gene sequence data. Red and blue symbols indicate the mean values (n = 3) for reactors 1 and 2, respectively. Error bars denote standard deviations. For AOB and NOB, only one taxonomic group (*Nitrosomonas* and *Nitrospira*, respectively) was identified by QIIME.

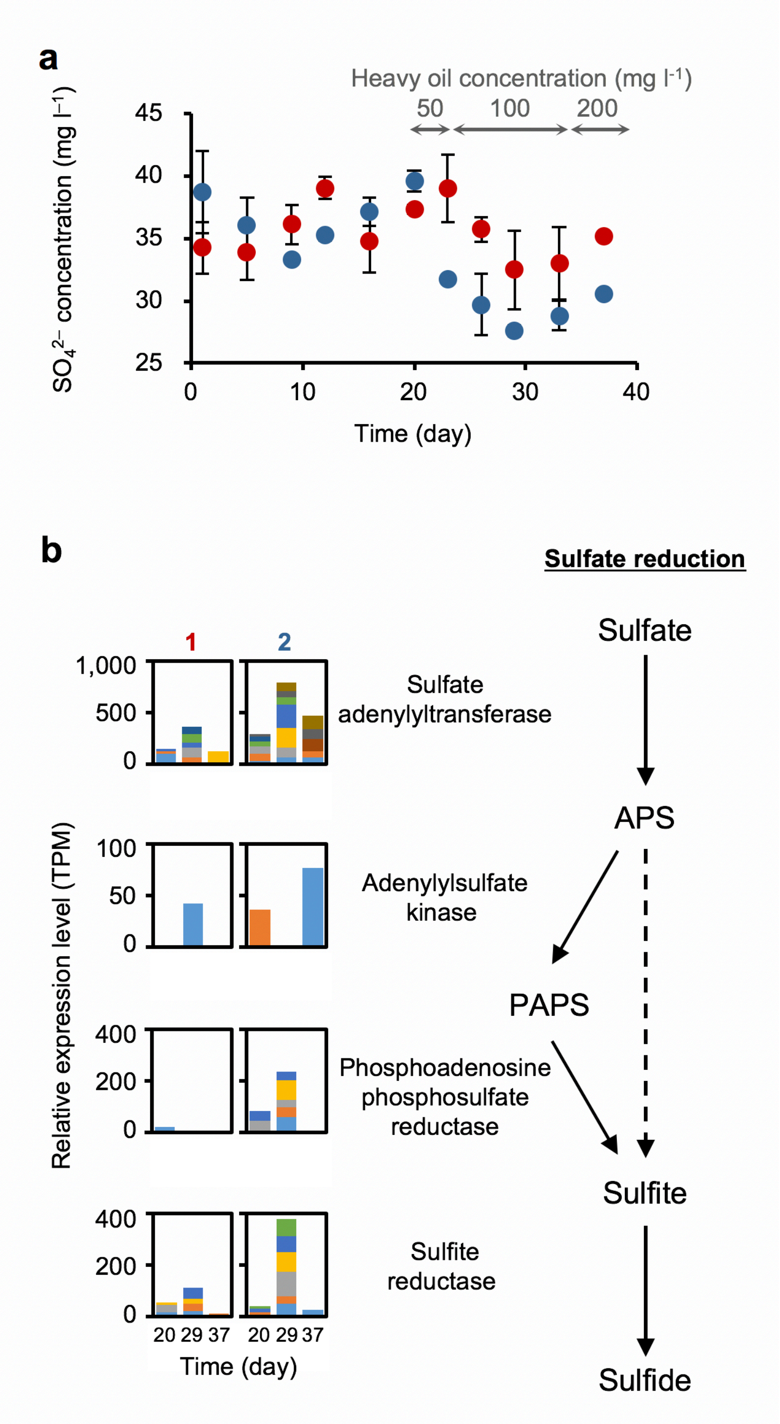

Supplementary Figure 9. Changes in sulfate concentration and expression of genes encoding sulfate reduction enzymes. a, Changes in concentration of sulfate. Red and blue symbols indicate the mean values (n = 2) for reactors 1 and 2, respectively. Error bars denote ranges. **b,** Relative expression levels of genes encoding enzymes in the sulfate reduction pathway: left and right panels correspond to the levels in reactors 1 and 2, respectively. Color-coded fractions indicate relative expression levels of individual transcript assemblies. Dotted arrow denotes dissimilatory sulfate reduction pathway that was not detected in this study. APS, adenylyl sulfate; PAPS, 3'-phosphoadenylyl sulfate.

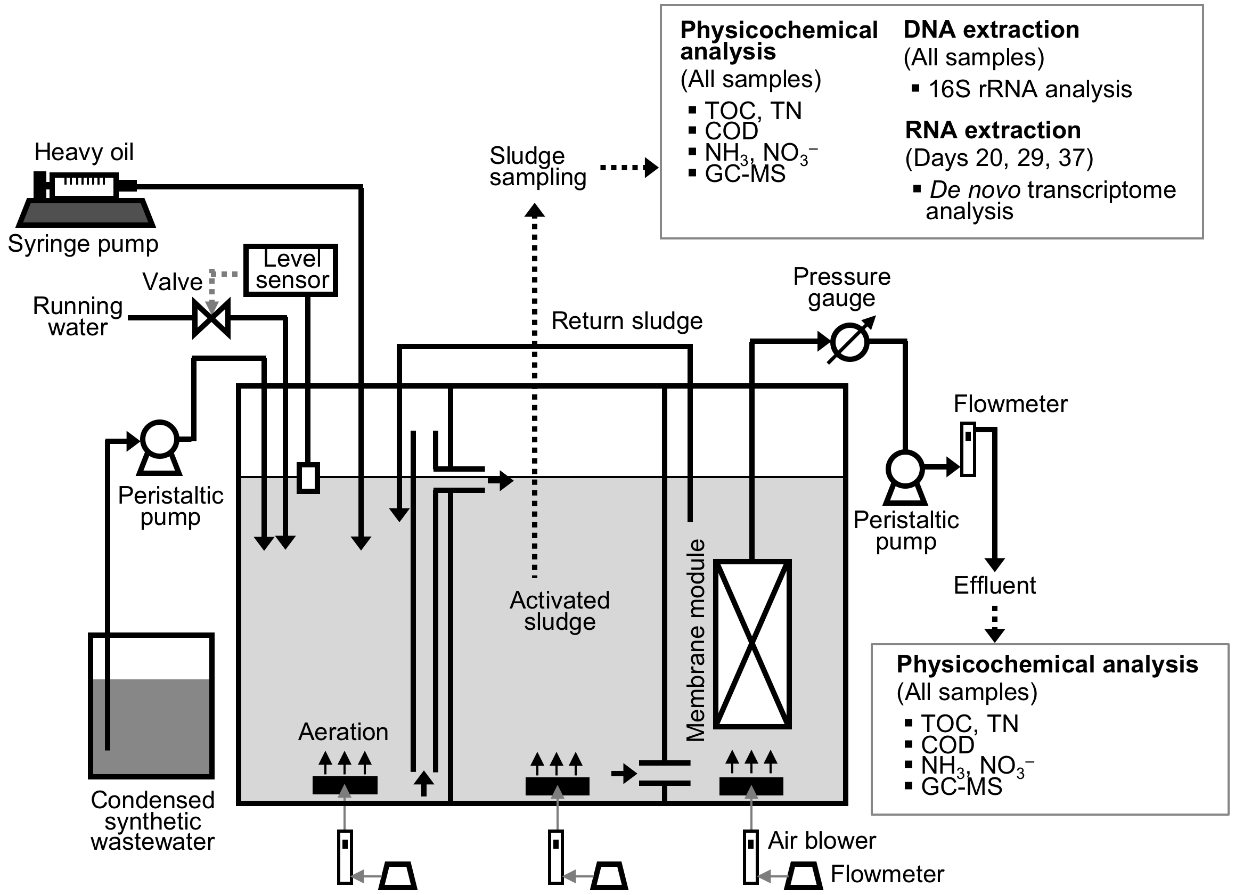

Supplementary Figure 10. Schematic configuration of the membrane bioreactor (MBR). The total working volume of the MBR was 230 l. The flow rate of influent synthetic wastewater, return sludge, and effluent was 115 l day^{-1} (hydraulic retention time = 2 days).

Supplementary Table 1. Summary of sequence analysis

		Reactor 1			Reactor 2		
	Time point	Day 20	Day 29	Day 37	Day 20	Day 29	Day 37
Raw reads and mapping ratio[a]	Total reads	7,979,418	7,766,914	6,228,468	3,642,478	3,660,073	2,604,746
	Reads after trim	3,508,353	3,362,763	3,605,484	1,460,332	1,574,216	1,827,531
	% Mapped to assembly	69.55%	77.11%	73.43%	70.60%	80.12%	73.78%
	% Mapped (before trim)	54.62%	64.01%	64.59%	54.57%	61.89%	66.49%

			Reactor 1	Reactor 2
Number of assemblies[c]		Total[b]	23,709	20,677
	Function assigned[d]	CDS (RefSeq, blastx)	20,612	17,976
		Functional	16,600	15,071
		Hypothetical	1,001	725
		Not assigned	3,011	2,180
		rRNA [6, 18, 23, 28S] (Silva, ribopicker)	2,686	2,371
		5S rRNA (5S rRNAdb, Fasta36)	6	5
		tRNA (tRNA scan)	85	74
		tmRNA (tmRNA db, Fasta36)	320	251

		Reactor 1		Reactor 2	
	Database	Function assigned	Not assigned	Function assigned	Not assigned
Number of coding sequence[e]	RefSeq (blastx)	17,601	3,011	15,796	2,180
	EggNOG (blastx)	5,192	15,420	5,075	12,901
	GO (blastx)	3,079	17,533	3,197	14,779
	Pfam (pfam scan)	3,431	17,181	3,403	14,573

[a]Total reads generated from Illumina sequencing were quality-filtered by using Trimmomatic. The reads before and after trimming were mapped to the assemblies by using Bowtie2.

[b]Total assemblies were generated by using Trinity[9].

[c]Sequences obtained at the three time points were merged into one. Reactor 1: a total of 10,476,600 sequences (3,508,353, 3,362,763 and 3,605,484 for days 20, 29 and 37, respectively) were used;

Reactor 2: a total of 4,862,079 sequences (1,460,332, 1,574,216 and 1,827,531 for the above days, respectively) were used.
[d]Functions of assemlies were assigned by searching various reference databases. Row names indicate type of transcript or database or program used.
[e]Row names indicate database or program used.
