# Supplementary material for: Transcriptome analysis of activated sludge microbiomes reveals an unexpected role of minority nitrifiers in carbon metabolism
Source: Commun Biol. 2019 May 13;2:179. doi: 10.1038/s42003-019-0418-2 (PMC6513846; doi:10.1038/s42003-019-0418-2)
Supplement: Supplementary file 3 — Description of Additional Supplementary Files [file 42003_2019_418_MOESM3_ESM.docx]

**Description of Additional Supplementary Files**

**Supplementary Data 1**

Source data for Figure 2. The tabs in the Excel file correspond to panels of the figure, as labelled.

**Supplementary Data 2**

Source data for Figure 3. The tabs in the Excel file correspond to panels of the figure, as labelled.

**Supplementary Data 3**

Source data for Figure 4. The tabs in the Excel file correspond to panels of the figure, as labelled.

**Supplementary Data 4**

List of increasingly and frequently expressed genes.

**Supplementary Data 5**

Relative expression levels of genes encoding aromatic hydrocarbon degradation enzymes and oxygenases of unknown specificity in various host organisms.

**Supplementary Data 6**

Relative expression levels of alkylsuccinate synthase-like genes in various host organisms.

**Supplementary Data 7**

Mapping of RNA reads onto the *Nitrosomonas* genome.
